# Pneumonia caused by toxic epidermal necrolysis

**DOI:** 10.1002/rcr2.1046

**Published:** 2022-09-29

**Authors:** Dominic Doyle, Amy Long, Desmond M. Murphy

**Affiliations:** ^1^ The Department of Respiratory Medicine Cork University Hospital Cork Ireland

**Keywords:** ciprofloxacin, pneumonia, toxic epidermal necrolysis

## Abstract

A well‐functioning 68 year old gentleman presented to our hospital with a macular rash 2 weeks after starting a course of Ciprofloxacin. There was rapid progression of skin involvement including the mucosa, complicated by pancytopaenia. Toxic Epidermal Necrolysis (TEN) was suspected and the patient was administered intravenous immunoglobulins and granulocyte colony stimulating factor. TEN was confirmed on skin biopsy and a lymphocyte transformation test demonstrated sensitisation to Ciprofloxacin. The patient developed multifocal pulmonary infiltrates with evidence of pulmonary involvement and probable pneumonia after 1 week and was treated with broad spectrum antibiotics. He also became dysphagic and suffered recurrent aspiration pneumonias. Follow up studies revealed fixed airways obstruction and features of bronchiolitis on computed tomography. This case highlights pulmonary involvement which can become a chronic complication of TEN, itself precipitated by the rare drug cause of Ciprofloxacin.

## Graphical abstract

We describe infective pneumonia secondary to disruption of the bronchial epithelium from a severe case of Toxic Epidermal Necrolysis Syndrome (TENS). This syndrome was due to Ciprofloxacin which is a very rare cause. Our patient recovered well with conventional treatment of TENS and pneumonia.

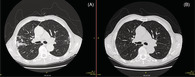

## INTRODUCTION

TEN is a rare condition that may be perceived as solely involving the skin but in fact can manifest in multiple organs. Our case describes pulmonary involvement with multifocal pneumonia, an acute pulmonary complication of TEN, with impaired diffusion capacity at 10 month follow up. The patient made an excellent recovery after a prolonged admission with targeted antibiotics and intravenous immunoglobulins (IVIg).

## CASE REPORT

A 68 year old gentleman was admitted to hospital with a 1 day history of sore throat, abdominal and testicular pain, and a macular rash on the face. He had a background history of smoking, colon and prostate cancer, and an artificial urinary sphincter. Regular medications included Amlodipine, Esomeprazole, Ferrous Fumarate, Atorvastatin, Tolterodine and Lisinopril/Hydrochlorothiazide; he had no known drug allergies. Two weeks prior to presentation he was administered a course of Ciprofloxacin. He had taken Diclofenac and Codeine phosphate 1 day before presentation, and had also been commenced on Tramadol 5 weeks prior to presentation. He deteriorated acutely with rapid progression of the rash to involve 90% of the skin, with oral and genital mucosal involvement. He was febrile, tachycardic and hypoxic. A clinical diagnosis of Toxic Epidermal Necrolysis (TEN) was made with a SCORETEN score of 4 (points for age >40, comorbid cancer, serum urea >10 mmol/L and >10% body surface area detached) indicating a 58.3% mortality rate. A skin biopsy was taken from the abdomen and histology confirmed the diagnosis showing sloughing of the epidermis with variable necrosis of keratinocytes, focally amounting to full thickness necrosis. C‐reactive protein peaked at 293 mg/L. Tramadol, Ciprofloxacin, Diclofenac, and Codeine phosphate were discontinued immediately. A lymphocyte transformation tests was performed across all of these medications and found type 4 sensitisation to Ciprofloxacin. He was commenced on IVIg on the second day of admission. Other therapies included intravenous hydration, opioid analgesia, nasogastric feeding, temperature maintenance and lubricant and steroid eye drops.

**FIGURE 1:**
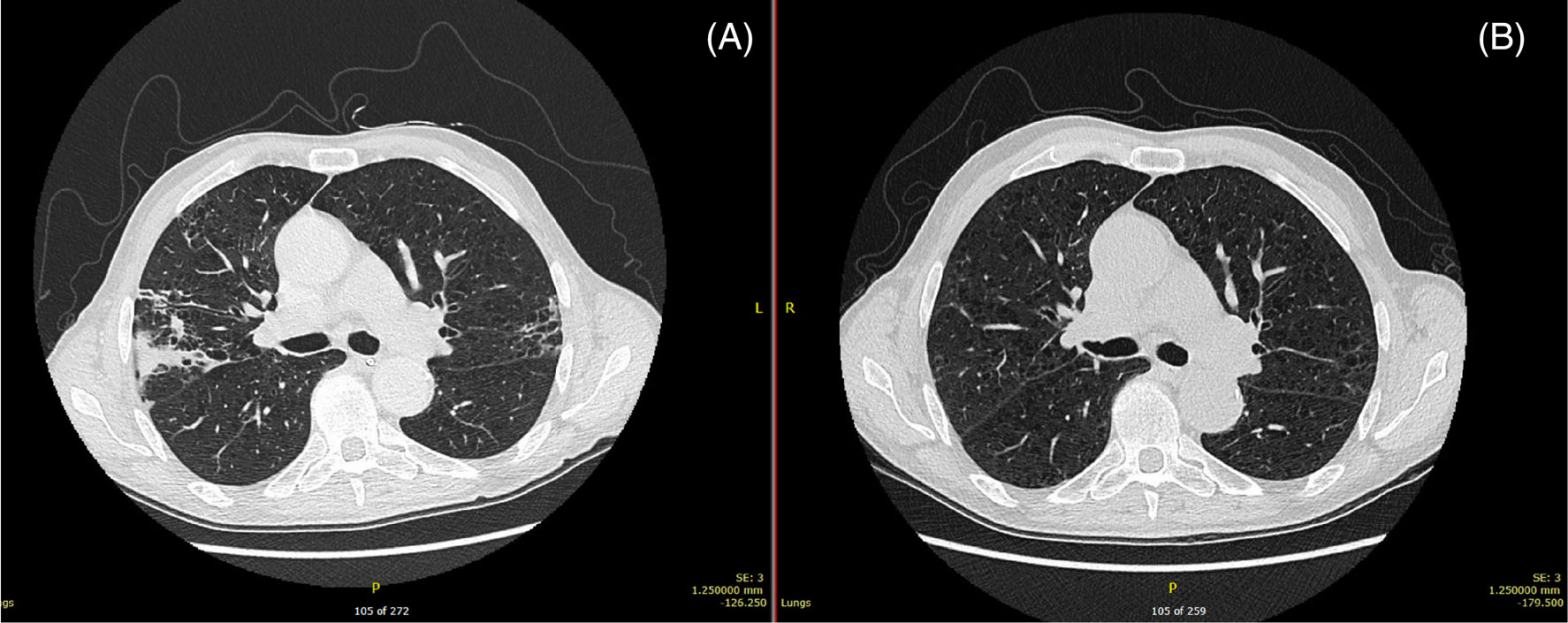
(A) Axial CT slice of multifocal pneumonia and (B) resolution

**FIGURE 2:**
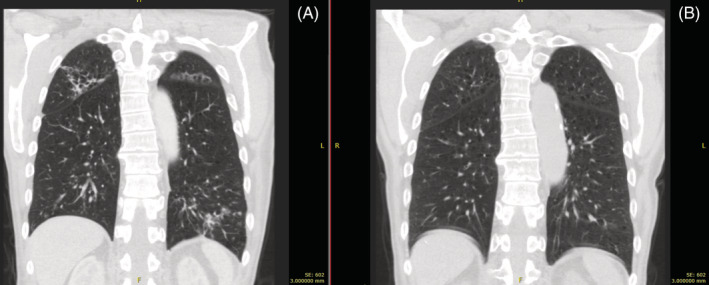
(A) Coronal CT slice of multifocal pneumonia and (B) resolution

He developed many complications over the following days most notably respiratory failure with evidence of evolving pulmonary involvement with progression to right upper and left lower lobe pneumonia. Sputum cultured *Escherichia coli* sensitive to Meropenem and Gentamicin, which the patient responded well to. He did not require mechanical ventilation. Immunoglobulin, autoimmune and rheumatoid screens were normal. He subsequently became pancytopaenic and was treated with granulocyte colony‐stimulating factor. A urinary catheter was required for the extended hospital admission and later converted to a suprapubic catheter. He had an upper gastrointestinal bleed and required transfusion of red cell concentrate. Furthermore, he developed severe dysphagia necessitating a percutaneous endoscopic gastrostomy for a total of 6 months. A follow up CT thorax 5 months later found almost complete resolution of the mucous plugging and tree‐in‐bud nodularity (see Figures 1 and 2). Subsequent pulmonary function testing showed a reduced FEV1/FVC ratio of 68% with mildly reduced FEV1 of 2.76 L (85%) and no reversibility. DLCO was reduced at 54%. Total lung volume was raised, measuring 8.6 L (119%) with evidence of air trapping (RV/TLC 129%). He uses a LABA/LAMA inhaler and has ongoing follow up for resultant aspiration pneumonias and exertional breathlessness although he maintains an active lifestyle.

## DISCUSSION

TEN is a severe mucocutaneous disease characterized by detachment of the epidermis and mucous membrane.^1^ It is a life‐threatening condition with a 30% mortality rate. It is typically drug‐induced and common causative agents include beta‐lactam or sulphonamide antibiotics, anti‐convulsants and non‐steroidal anti‐inflammatory drugs. Other less common causes include immunization, Mycoplasma pneumoniae or Cytomegalovirus infection, graft versus host disease and contrast medium. Bronchial epithelium can be affected by TEN and become detached leading to acute and, sometimes, chronic complications. Furthermore, superimposed infection may occur. One early prospective study of 41 patients with TEN found that a quarter had acute pulmonary involvement with bronchial mucosal sloughing on bronchoscopy. Dyspnoea and sputum production were common features yet, of 10 affected patients, 8 had normal plain film chest imaging.^2^

A literature review examining chronic pulmonary complications in TEN and Stevens‐Johnson syndrome (SJS) identified 22 cases, of which 10 exhibited bronchiolitis obliterans. Interval onset was from weeks to months and is likely a consequence of damage to the bronchiolar epithelium and alveolar membrane. Bronchiectasis and bronchitis were the next most common pathologies, respectively. Steroids and bronchodilators were the mainstay of treatment modalities employed and survival was variable but generally poor with seven patients succumbing within 2 years.^3^ Conversely, another study following up pulmonary function testing in 32 patients revealed moderately reduced diffusion capacity in 13 (41%) patients at approximately 3 months but no obstruction; moreover most of these patients were asymptomatic. Patients with impaired diffusion capacity tended to have a greater burden of skin detachment at presentation. Six of 13 patients had a smoking history and 3 had radiological pulmonary infiltrates. Eight of 10 patients who had repeat pulmonary function testing at a median time of 12 months had persistently reduced diffusion capacity.^4^

The optimal management of TEN has not yet been universally established. Of course, discontinuation of the offending drug is a widely accepted measure along with good supportive care.^5^ However, beyond this, there is a paucity of evidence. Immunomodulating therapies including systemic corticosteroids, IVIg, Cyclosporin, plasmapheresis and anti‐tumour necrosis factor monoclonal antibodies have been used in practice but have been inadequately studied thus far. We propose managing pneumonia and chronic complications along standard guidelines. In this case, it was initially unclear which of the newly prescribed drugs caused TEN. Ciprofloxacin proved to be the culprit and this fluoroquinolone is a rare cause of TEN. To our knowledge only eight case reports have documented this association, and our report is the only one where lymphocyte transformation testing has been used confirming sensitisation to the drug. This case report supports the preceding evidence, further highlighting the potentially life‐threatening drug reaction to Ciprofloxacin. Acute pulmonary involvement in TEN is not uncommon and early respiratory symptoms with hypoxemia despite a normal chest X‐ray may indicate pulmonary disease. Mild pulmonary dysfunction as a long‐term sequela may be underappreciated.

## AUTHOR CONTRIBUTIONS

**Dominic Doyle**: Corresponding author. **Amy Long**: Co‐author. **Desmond Murphy**: Attending Consultant and Supervisor.

## CONFLICT OF INTEREST

None declared.

## ETHICS STATEMENT

The authors declare that appropriate written informed consent was obtained for the publication of this manuscript and accompanying images.

## Data Availability

The data that support the findings of this study are available on request from the corresponding author. The data are not publicly available due to privacy or ethical restrictions.
